# Advertising Violent Toys in Weekly Circulars of Popular Retailers in the United States

**DOI:** 10.15171/hpp.2015.023

**Published:** 2015-10-25

**Authors:** Corey H. Basch, Laura A. Guerra, Rachel Reeves, Charles E. Basch

**Affiliations:** ^1^Professor, Department of Public Health, William Paterson University, Wayne, NJ 07470, USA; ^2^Department of Health and Behavior Studies, Teachers College, Columbia University, New York, NY 10027, USA

**Keywords:** Toys, Violence, Role socialization, Gender

## Abstract

**Background:** Violence is a pervasive problem in the United States. Toys, far from trivial playthings, are a reflection of society, including its beliefs and values. The purpose of this study was to describe the extent to which violent toys are marketed in online weekly flyers of popular retailers, how the violence is manifested, and whether violent toys are marketed differentially to boys and girls.

**Methods:** For this cross-sectional observational study, online circulars from 5 major retailers were downloaded and examined each week for 14 weeks during the fall of 2014. For each retailer, the total number of toys, as well as the total number of violent and non-violent toys, was recorded. In addition, each violent toy was categorized into one of five groups: picturing a figure with a weapon, a figure with intent to strike (with fists drawn or an angry face), a toy with a violent name, a toy that was a weapon itself, or a set of toys that included two or more of these criteria.

**Results: ** A total number of 3,459 toys were observed, of which 1,053 (30%) were deemed violent. Of the violent toys, 95% were marketed to boys (n=1,003) versus 5% to girls (n=50). The most prevalent violent category was a figure with a weapon such as a sword, knife or gun (29%), followed by figures with fists out and aggressive faces (26%).

**Conclusion:** Parents should be mindful of toy retailer‟s marketing of violent toys, especially toward boys, and the potential for those toys to de-sensitize their children to violence

## Introduction

Interpersonal violence is one of the biggest public health problems across the globe, according to WHO.^1^ Reports on the global impact of violence have revealed that tremendous gaps in reporting and treatment grossly underestimate the burden violence places on society.^1^ The United States (U.S.) Department of Justice has estimated that only 50% of violent crimes are routinely reported.^2^

Nevertheless, the United Nations Office on Drugs and Crime indicated that 437,000 people around the globe were killed in 2012 as a consequence of intentional homicide.^3^ Men are most commonly affected, accounting for 80% of homicide victims and 95% of perpetrators.^3^ Domestic violence remains an important cause of homicide, accounting for 15% of all cases; 70% of domestic violence victims are women.^3^

In the U.S. in 2012, the age-adjusted mortality rate due to violence was 19.63 per 100,000 population.^4^ Compared with every other age group, youth between the ages of 15 and 24 are at the highest risk for becoming a victim of murder.^5^ Globally, the homicide rate among males is more than three times greater than females (9.7 per 100,000 versus 2.7 per 100,000).^3^Homicide is most prevalent among males residing in the Americas, with a rate of 29.3 per 100,000 males.^3^ In the U.S., intentional acts of violence claim the lives of roughly 50,000 individuals annually.^5^ In 2013, over 16,121 people were victims of homicide (11,208 from firearms), and 41,149 succumbed to suicide.^6^

A current report by the Bureau of Justice Statistics declared that the rate of violent victimization among youth aged 12 and older increased by over 15% from 2011 to 2012 (22.6 victimizations per 1,000 versus 26.1 victimizations per 1,000).^7^ Rates for nonfatal injuries are highest in young adults aged 15 to 24.^8^ Physical assault resulted in the need for emergency hospital attention for half a million youth aged 10 to 24 in 2013.^8^ It is estimated that the cost of youth violence is roughly $160 billion per a year.^9^

Researchers in Chicago have studied the effect of neighborhood violence and found that children who lived within 1,000 feet of a recent homicide crime scene experienced a significant decline in test scores, attention levels, and impulse control.^10^ Directly witnessing domestic violence as a toddler has been shown to predict reduced memory capacity later in childhood.^11^ Exposure to and being a victim of violence during childhood has been associated with increased morbidity and mortality due to chronic heightened inflammatory responses.^12^ The economic burden of violence has been estimated to equal 3.3% of the gross domestic product of the U.S.,^13^ but could be much higher considering days of lost productivity.

Violence is not a problem that any one change can solve. While some argue that the perpetual cycle of victims becoming perpetrators and a lack of uniform support systems may explain the majority of violent incidents,^1^ childhood exposure to violence and learned behaviors have been shown to predict hostility later in life.^14^In order to tackle the pervasive nature of violence in the U.S., significant societal change must occur to address the problem’s multifactorial etiology.

In the past decade, there has been a growing focus on the effect of violent television on the developing child,^14^ but little research has been done to identify the role that violent toys can play in intellectual development. Because playing with an item, long considered an active action, differs inherently from the passive process of viewing programming, toy play significantly contributes to psychological growth and advancement.^15^ As cited in Jenvey, The Victorian Ministry of Consumer Affairs Special Committee of Inquiry into Victim Toys has concluded that violent and anti-social-type toys do have the potential to induce psychological harm in young children.^15^

Toys, far from trivial playthings, are a reflection of society, including its beliefs and values. Research suggests that during early history, a toy’s chief role was to convey moralistic messages. Over time, toys took on additional roles, functioning as both a recreational outlet and an opportunity to learn desirable skills or those necessary for adulthood. Toys have been observed as a means to convey status and a mechanism to cultivate attitudes or beliefs.^16^

One way that parents encourage and reward the ‘sex-appropriate’ socialization of their children is buying gender-typed toys.^17^ A number of behavioral differences between females and males, including problem solving, impulsiveness, and aggression, have been attributed to differences in socialization.^17^Boys may be the primary targets of aggressively oriented social content, and overtime, may become less sensitized to violence than their female counterparts.^18^ Other studies suggest that exposure to violent television and video games may be correlated with higher rates of physical aggression among children.^19-21^

Rates of violent crime arrests among teen and young adult males have declined by nearly 50% over the last two decades,^22^ but violence remains pervasive in the lives of American youth. Almost one in five American high school students (grades 9-12) were involved in a physical altercation within the last 12 months (30.2% of males and 19.2% females).^23^ A significant proportion of these students (5.6% of whites, 7.9% of Blacks, and 9.8% of Hispanics) missed one or more school days per month because they were afraid to be at school or to travel to or from school (and these rates have been increasing).^23^

Video games that display violence now require age appropriate ratings. However, toys marketed to toddlers and young children encourage the use of weapons and fighting, and the marketing of these toys remains unregulated.^24^ Although war toys have traditionally been among the best-selling categories of children’s playthings,^25^ concern for their ability to desensitize children to violence is not new.^26^ Despite trepidation expressed by some in medical^27^and academic^28^ communities regarding toy gunplay, many parents are convinced that such play leads to increased violence.^26,28^ Such parental views vary by gender of the child, gender of the parent, and race.^24,28^

We did not identify any published reports describing the extent to which toys associated with aggression were marketed to children. We therefore observed and coded online weekly flyers from popular retailers to describe the extent to which toys associated with aggression and violence were marketed to children, and whether there were differences in the frequency with which such toys were marketed toward boys versus girls.

## Materials and Methods

For this cross-sectional observational study, online circulars from five major retailers based in the U.S. were downloaded and examined each week for 14 weeks during the fall of 2014 (September 21 to December 21), a time period when holiday celebration encourages the purchase of children’s toys. Retailers included were members of the National Retail Federation’s top 100 retailers of 2014.^29^ Stores were chosen based on carrying children’s toys and advertising them in their weekly sale circulars. One store was a large chain retailer of children’s toys, while three were highly popular chain discount retailers, and the final entity was a large department store chain that has a toy section that is marketed. All retailers maintained a physical store location no more than 15 miles from the location of data collection as well as a strong national presence. Two stores included in this sample sustain an international presence as well. Together they maintain 10,448 stores across the U.S.^30^ The global information company National Purchase Diary estimates retail sales of toys generated $18 billion in revenue in 2014.^31^

Sixty-six circulars were included in this sample. It should be noted that, due to double issues (those that spanned two or more weeks) throughout holiday periods, some retailers did not publish a new circular for each of the 14 weeks. For each retailer, the total number of toys, as well as the total number of violent and non-violent toys, was recorded. In addition, each violent toy was categorized into one of five groups: picturing a figure with a weapon, a figure with intent to strike (e.g., fists drawn or an angry face), a toy with a violent name or wording, a toy that was a weapon itself, or a set of toys that included two or more of these criteria. Of the violent toys, the gender the toy was marketed to was determined by analyzing the name and appearance of the toy. Toys typically marketed toward boys that now appeared in gender specific color and design (e.g. a pink gun with heart decorations), were determined to be marketed toward young girls. Two of the five retailers advertised boys and girls toys on separate pages consistently for each sale circular. Violent toys were very clearly divided among gender lines, and no violent toy was determined to be sex-neutral.

To demonstrate the consistency of coding, violent toys were classified and coded by one researcher (RR); 10 were re-coded by two researchers (CHB and RR). Cohen’s kappa was used to assess inter-rater reliability and was found to be excellent (k = 0.91).

“Toys” were defined as any tangible play-item marketed to children for the purpose of entertainment. Toys included in the study ranged from dolls and action figures to board games and sports equipment (e.g. basketball, basketball hoop, etc.). Electronic media such as video games and children’s educational tablets were excluded from both the numerator and denominator based on software uncertainty.

Toys that were sold separately were counted as two different items. For example, if a doll was pictured in both a blue and a pink dress, they were counted as two different toys. If these same two dolls were listed as a package of two, they were counted as one item. Sets of action figures with multiple characters exhibiting both fists drawn and weapon use were characterized as a set. Items that used weapons and had a violent name were characterized as a set as well.

Data analysis methods utilized descriptive statistics, including calculations of frequencies, percentages, means and standard deviations. Analyses were performed using IBM SPSS (version 22).

### Ethical Considerations

This study was determined not to be human subject research by the Institutional Review Board at William Paterson University. Studies that do not involve human subjects are not reviewed by the Institutional Review Board​s at William Paterson University or Teachers College, Columbia University.

## Results

A total of 3,459 toys were observed throughout the circulars, of which 1,053 (30%) were considered to be violent. Four out of five of the retailers observed had approximately one third of their online circular devoted to violent toys. Among the five retailers, the mean number of toys included in each online circular was 53.2 (SD = 66.9). Of the violent toys, 95.3% percent (n=1,003) were marketed to boys (Table 1). Across the retailers, the average number of violent toys marketed to boys per circular was 15.43 (SD = 20.37). The most prevalent category of violence was a figure with a weapon such as a sword, knife or gun (29%), followed by those figures with fists out and aggressive faces (26%), and the product itself being a weapon (16%). Less frequently noted categories of violence included a game or product with violent wording (6%). It should be noted the remaining 23% was a toy set with multiple violent categories.

**Table 1:** Total number and percent of violent toys by retailer during the 14-week observation period

**Retailer**	**Total Toys in Circulars**	**Total Violent Toys in Circulars n**	**Violent Toys in Circulars (%)**	**Multiple Violence Categories n (%)**	**Figure with Weapon** **n (%)**	**Figure with Raised Fist, Aggressive Face** **n (%)**	**Violent Wording** **n (%)**	**Toy as Weapon** **n (%)**	**Violent Toy Marketed Toward Boy** **n (%)**
1	1353	424	31	116 (27)	128 (30 )	113 (27)	20 (5)	47 (11)	408 (96 )
2	337	69	20	7 (10)	18 (26)	16 (23)	6 (9)	22 (32)	61 (88)
3	561	155	28	27 (17)	42 (27)	40 (26)	14 (9)	32 (21)	148 (95)
4	670	231	34	48 (21)	66 (29)	59 (26)	19 (8)	39 (17)	219 (95)
5	538	174	32	43 (25)	53 (30)	44 (25)	8 (5)	26 (15)	167 (96)
All stores	3459	1053	30	241 (23)	307 (29)	272 (26)	67 (6)	166 (16)	1003 (95)

## Discussion

To our knowledge, this is the first study to focus on violent toys promoted in weekly circulars of major national retailers. Our findings revealed that violent toys were regularly promoted. Nearly all of the violent toys in the weekly circulars were marketed to boys. Morbidity, mortality, disability due to violence, and consequential intentional injuries are pervasive problems in the United States.^4^ Violence remains a critically important issue for youth,^7,8^ with far ranging consequences spanning from injuries and hospitalizations^8^ to missed school days and living in fear.^22,23^ Experience with violence differs for subsets of the population. For example, homicide rates are far greater in males than females.^3^ The degree to which this gender discrepancy is a result of socialization must be considered.

The implications of playing with violent toys are unclear for child and adolescent development. Nevertheless, it is clear that many behaviors learned in childhood persist into adolescence.^32^ Children learn by watching others and imitating behaviors. Because children’s programming oftentimes contains a significant amount of violence, the American Academy of Pediatrics advises parents to discourage children from playing with television-related toys where the child has the opportunity to mimic the violence they may see ‘acted out’ by their favorite characters.^24^ We believe the same type of prudent advice is warranted in parental purchasing of violent toys. This is of particular concern since advertising and marketing is commonly either directly or indirectly aimed toward children. Children tend to think of their toys as extensions of themselves.^33^ To the extent this occurs, there is great potential for children to imitate violent toy play and equate the capabilities of their playthings as representations of normal behavior.

There is a paucity of published research on how advertisements and promotions affect parents’ toy purchasing behavior. Research on food purchasing behavior suggests that youth influence parents’ decisions.^34^ The extent that this also applies to the purchase of toys is an area for future research. Regardless, the consistent promotion of violent toys week to week by all the retailers suggests there is a viable market for such toys. Parents who wish to avoid these toys should seek to mitigate the effects of all such marketing, including helping children to understand the destructive toll that real weapons can take on families. Parents may also use such discussions to develop their children’s awareness of the emotionally manipulative nature of advertising.^35^ Other strategies include establishing rules for what constitutes an appropriate toy, and discussing such rules with their children.

This study is limited in that it was cross-sectional in design and included only 14 weeks of circulars from 5 retailers. Future studies could focus on a longer period with additional retailers. Despite these limitations, this study fills a gap in current knowledge. Clearly, violent behaviors are learned in a variety of contexts and reflected in a variety of ways in society. To the extent that parental purchasing of violent toys influences child and adolescent behavior, this could be an important opportunity for public health education by government agencies concerned with violence prevention in schools and family education forums. In addition to targeted individual changes, societal changes should be considered as well. For example, health advocacy may affect policymakers and legislators in labeling toys as violent.

## Conclusion

Parents should be mindful of toy retailer’s marketing of violent toys, especially toward boys, and the potential of those toys to de-sensitize their children to violence.

## Acknowledgements

The authors declare that there is no conflict of interests. There was no funding for this study.

## Conflict of Interests

The authors report no conflict of interest.
